# The effect of protein supplements on weight loss, body composition, protein status, and micronutrients post laparoscopic sleeve gastrectomy (LSG): A Randomised Controlled Trial (RCT)

**DOI:** 10.1016/j.amsu.2021.103220

**Published:** 2022-01-01

**Authors:** Sahar Alshamari, Mohamed Aly Elsherif, Fahad Hanna, Leyan El Akhal, Hana Abid, Wahiba Elhag

**Affiliations:** a Department of Bariatric Surgery/ Bariatric Medicine, Hamad General Hospital, Hamad Medical Corporation, Doha, Qatar; b Department of Public Health, Program of Public Health, Torrens University, Australia

**Keywords:** Laparoscopic sleeve gastrectomy, Protein supplement, Body composition, Muscle mass, Fat mass, Protein deficiency, Micronutrients deficiency

## Abstract

**Background:**

Low protein intake post-bariatric surgery can result in protein malnutrition, and muscle mass loss. Authors aim to assess the effect of protein supplements on weight loss, body composition, and micronutrient status following LSG.

**Methods:**

This is a double-blinded RCT conducted between February/2017 to January/2018. Eligible post LSG patients were randomized into the intervention group who received daily protein supplements containing 20 g of protein and placebo group received zero protein supplements. Both groups received a standardized diet. Weight loss, body composition, and micronutrient status were analyzed at 1, 3, and 6 months.

**Results:**

48 participants were included in the final analysis (intervention: 21 and placebo:27). Excess weight loss percentage (EWL%) at 6 months was comparable between both groups (69.44 ± 21.99% and 71.40 ± 19.27% respectively). No significant difference observed in the anthropometric parameters. There was an increase in muscle mass and a decrease in muscle mass loss in the intervention group throughout the study period. However, these changes were not statistically significant. There was a significant increase in total protein (*P=0.027)* and magnesium (*P=0.008*) in the intervention group at 3 months. Albumin and iron levels were significantly higher at 6 months in the intervention group (*P=0.036* & *P=0.028* respectively). Other micronutrients did not differ at any time point between both groups.

**Conclusion:**

Protein supplements resulted in significant improvement in total protein, albumin, magnesium, and iron levels post LSG. Although not significant, protein supplements helped in maintaining the muscle mass and preventing muscle mass loss.

**Original article:**

This RCT is an original article and provides a level 2 evidence.

## Highlights

• Low protein intake post-bariatric surgery results in protein malnutrition & muscle mass loss. Protein supplements may be of benefit.
• Eligible post LSG patients were randomized into the intervention group who received daily 20 g protein supplements & placebo group.
• No significant difference in weight, BMI, BMI change, EWL%, TWL%, and absolute weight loss. No statistically significant changes in the muscle mass.
• Percentage of muscle mass loss was noticeably higher in the control group over the 3 timelines. Fat percentages were less in the intervention group.
• Total protein and Albumin showed a significant increase in the intervention group.

## 1 Introduction

Laparoscopic Sleeve gastrectomy (LSG) has gained great popularity due to its simplicity and excellent outcomes in terms of weight loss and improvement of obesity-related comorbidities [[1], [2], [3]]. The alteration of the anatomical structure of the gastrointestinal tract post LSG results in the desired weight loss metabolic and effects. Despite these great benefits, undergoing LSG may compromise the nutritional status due to restriction of energy and protein intake with subsequent protein and micronutrient deficiencies [[4], [5], [6]]. Such deficiencies can result in significant morbidity if not prevented including anemia and metabolic bone disease. On the other hand, the substantial and rapid weight loss especially in the first 6 months after LSG not only results in a fat mass loss but also is accompanied by loss of muscle mass. Loss of muscle mass may be undesirable when excessive muscle mass is responsible for the majority of resting metabolic rate, regulation of core body temperature, preservation of skeletal integrity, and function [[7], [8], [9], [10]]. Therefore, to make bariatric surgery a safe and efficient procedure, the goal during weight loss should aim to maximize fat mass loss while preserving metabolically active muscle mass. To achieve this goal, adequate dietary proteins and balanced nutrition are needed. Current nutritional guidelines recommend an average daily protein intake of (90–120 g) or 1.1 g/kg of ideal body weight following LSG to reduce the undesirable lean muscle loss [11]. However, these recommendations are not conclusive [11,12], with a large proportion of LSG patients fail to comply with daily protein requirements due to the restricted food intake and malabsorption during the post-surgery period [13]. Other contributing factors are the occurrence of vomiting or regurgitation, intolerance to protein-rich foods, and poor eating patterns [[14], [15], [16]]. For those patients who fail to consume an adequate amount of protein and micronutrients in their diets, supplement is recommended to increase their protein and micronutrients intake [13,17]. The quality of the protein source is also very important, particularly the quantity of leucine, which helps maintain muscle mass. For this reason, protein supplement made of whey protein has been recommended as the choice of supplement for increasing leucine uptake [18]. Other measures essential to preserve muscle mass and preventing nutritional complications post-LSG also include regular monitoring, administration of multivitamins, and emphasis on regular exercise as the patient recovers [19,20].

Little is known about the effect of protein supplements post-bariatric surgery since most studies focused mainly on dietary protein intake [[21], [22], [23], [24]]. Most studies that assessed protein supplements were conducted post-Rou en Y bypass surgeries (RYGB) [25,26]. The few publications which evaluated the effect of protein supplements post LSG were observational studies [27,28], that included a small number of LSG patients and assessed limited micronutrient parameters [13,29]. To our knowledge, there is only one randomized control trial (RCTs) that addressed the influence of protein supplements post LSG [30], however, it combined two types of procedures i.e. LSG and RYGB that have different mechanisms of weight loss and varying effects on body composition [29,31]. The aim of this study was to evaluate effect of protein supplements on weight loss, body composition, and micronutrient status following LSG.

## 2 Hypothesis

In this RCT we evaluated the effect of protein supplementation at 1, 3 and 6 months post LSG on weight loss, body compositions, protein status and a range of micronutrients. We hypothesize that protein supplementation during the initial period of post-surgery would significantly reduce the impact of protein and other nutrients loss. We envisage that adherence and compliance in this trial population would further optimize the results of the bariatric surgery through fat loss and reduction of muscle mass. To our knowledge, this is the first RCT to assess the impact of protein supplements post LSG.

## 3 Materials and methods

### 3.1 Study design, ethics, and participants

This double-blinded RCT study was conducted in the period from 16/February/2017 to 15/January/2018. Ethical approval was obtained from the IRB committee, approval number (16433/16). This RCT trial was also registered before the commencement of the study with the US NIH ClinicalTrials.gov., registration number (NCT03147456).The study was also registered in https://www.researchregistry.com, registration number: (researchregistry7348). Informed consent was obtained from each participant before enrollment in the study. Participants with an indication for laparoscopic sleeve gastrectomy were consecutively enrolled in a routine clinical setting according to the American Society of Bariatric and Metabolic surgery (ASBMS) guidelines [12]. This trial was conducted in accordance with the CONSORT criteria (2010) and entire check list is submitted as an attachment.

### 3.2 Inclusion and exclusion criteria

Participants were enrolled in this trial according to the following criteria: age between 18 and 60 years, BMI ≥35 kg/m^2^ with comorbidities, or BMI ≥40 kg/m^2^ who are scheduled to undergo primary LSG. Patients with renal or hepatic disease and those with revisional bariatric surgeries were excluded from the trial. Participants who won't consume at least 80% of the supplements throughout the study period (minimum of 24 bottles/month) were excluded due to their poor compliance with the study protocol.

All eligible participants are patients from the bariatric surgery services at Hamad Medical Corporation, Doha, Qatar.

### 3.3 Randomization

A total of 100 participants were determined eligible and accepted to be enrolled in the study and were subsequently randomized to intervention or placebo groups. Microsoft Office Excel 2010 (Microsoft Corporation, Redmond) was utilized to generate random numbers defining the block order. Due to our strict compliance policy, the number of patients included in the analysis was reduced. No intention to treat analysis was conducted.

### 3.4 Treatment

At the start of the trial, dietary advice was provided to both groups by bariatric dietitians. Verbal and written instructions were offered during hospitalization, and at 1, 3, and 6 months after surgery. Participants were advised to strictly follow a post-bariatric diet according to ASMBS Allied Health Nutritional Guidelines to ensure that both groups safely consume a similar diet [32]. Before discharge, the intervention group received supplement bottles “Cubitan ®, Protein, Nutricia, Netherlands” that contain 20 g of protein and 250 kcal. On the other hand, the participants in the placebo group were provided with identical supplement bottles “preOp ®^,^ Nutricia, Netherlands”, that contained no protein and 100 kcal. Both groups were advised to drink one bottle daily over 3–5 intervals. Each bottle was wrapped with identical packaging to enable the blinding. Participants in both groups were also provided by standard multivitamin supplements. A food frequency questionnaire was obtained upon each visit from each participant. Physical therapists encourage participants to exercise for 150–300 min per week, starting with light to moderate activities.

### 3.5 Study procedure

Nutritional assessment and dietary counseling were scheduled with the bariatric dietician at 1, 3, and 6 months after LSG. At each study visit, participants in both groups were interviewed about the frequency and number of supplements consumed since the last consultation to determine their eligibility to be part of the study's final inquiry. Participants were encouraged to use a diary to detect use. Additionally, all participants were followed up through weekly phone calls to ensure compliance with supplement intake and adherence to the study protocol. They were also offered dietary advice promoting a hypocaloric protein-rich diet. Food frequency questionnaire was obtained at the 3- and 6-months follow up visits when participants transitioned from a semiliquid diet to solid food. Three food categories that were investigated included whole meat (Beef, Chicken, Fish), eggs, and dairy products as high protein food items, starchy food that included pasta, bread, grains and sweets, and finally, fruits and vegetable intake as a third food category. For each food category, the patient's intake was classified into low, moderate, or high. All questionnaires were administered in Arabic and English languages.

Blood samples for biochemical and micronutrient parameters were collected after 8 hours fasting, before surgery and at 1,3 and 6 months post-operatively.

### 3.6 Body composition assessment

Measurements were taken before LSG, and at 1, 3, and 6 months postoperatively. At each visit, body weights were assessed by (Seca® 869 flat digital scale) and body composition was measured after an overnight fast using bioelectrical impedance analysis (BIA; Tanita BC-418 MA, Body Composition Analyzer). Height was measured using a fixed wall stadiometer. %EWL, BMI change, absolute weight loss, muscle mass, and fat mass losses % were calculated using the methods described in previous studies [13,24,33].

### 3.7 Outcomes and data collection

Primary outcomes were weight loss and body compositions at 1, 3, and 6 months post-LSG. Variables assessed included weight, BMI, %EWL, weight loss, BMI change, muscle mass, fat mass, fat percentage, percentage of muscle mass loss, and percentage of fat loss. The secondary outcomes were the changes in proteins and micronutrients status at 1,3 and 6 months post LSG. Data collected was total protein, albumin, hemoglobin, iron, folate, vitamin B12, vitamin D, calcium, magnesium, zinc, and copper. We also compared the intake of whole meat, eggs, dairy products, starchy food, fruits, and vegetables between placebo and intervention groups at 3 and 6 months post LSG.

### 3.8 Endpoints

In our trial we focused on endpoints that are meaningful to patients, clinicians and decision makers where evidence can be used for improvement of healthcare in this group of patients. Reducing muscle mass loss and nutrients loss is crucial for various functions and indeed for the survival of these patients. We also ensured these endpoints are not only valid but also can be objectively measured in this population. This was achieved through validated and reproducible measures performed by trained and experienced clinical personnel. ***Sample size calculation and Statistical Analysis***.

Calculation of sample size was done based on the fact that the estimation of 44 patients per group would provide 84% power and 5% probability of type I error. This was principally based on the primary and secondary outcomes of this study. Assuming a 50% dropout rate, target enrollment was set at 88 per group. The actual dropout rate was 43%, which is close to the large pharmacological weight management trials. Quantitative variables were expressed as mean ± standard deviation. To observe the changes in anthropometric, body composition, and biochemical parameters between groups, an independent *t*-test, and Mann Whitney test was applied, if otherwise, paired sample T-test and Pearson Chi-square test used for comparisons were used for intergroup comparison of categorical variables. Missing data were not imputed. All analyses were two-tailed. P-value ≤ 0.05 was interpreted as statistically significant. The raw data were entered using Microsoft Excel Sheet and analyzed using SPSS package 25, version 19.00. A block randomization procedure (www.randomizer.org) was employed (2 Sets of 50 Unique Numbers Per Set Range: From 1 to 100) to ensure the balance between the groups. The allocation ratio was 1:1. The randomization process including enrollment of participants and the assignments of participants to control groups was supervised by nursing staff who were not members of the research team.

## 4 Results

**Fig. 1:**
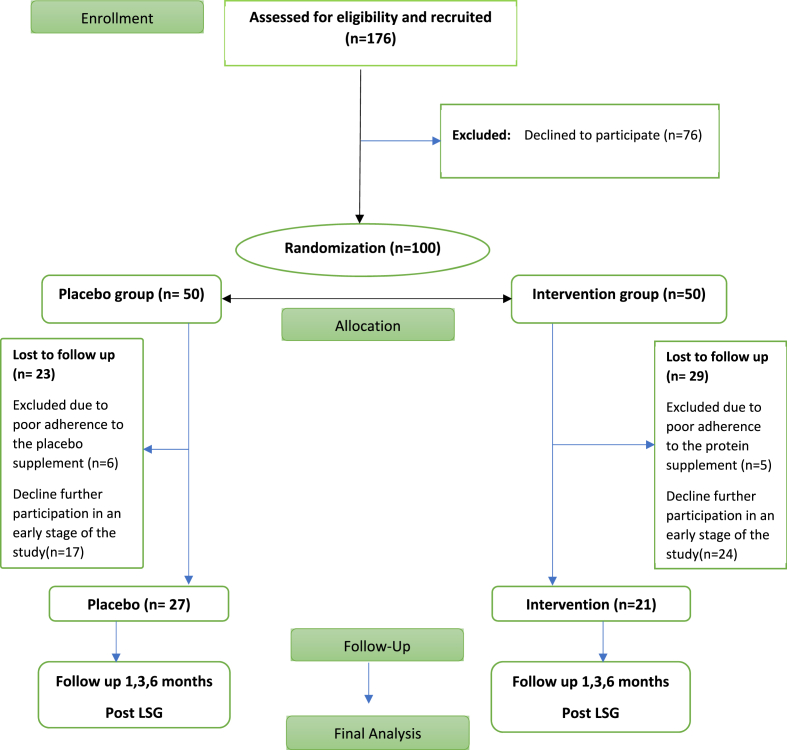
Flow chart of participants through the trial.

The flow chart of a total of 176 eligible participants is described in Fig. 1. Seventy-six patients had withdrawn and declined to participate in the study before the start of the intervention. The remaining 100 participants were randomized (50/50) into either the intervention or the placebo arm of the study. Twenty-nine participants from the intervention group and 23 from the placebo group were excluded due to noncompliance with the supplement consumption (<80% consumption) or declined further participation in the study. The remaining 48 participants (21) in the intervention group and (27) in the placebo group completed the study and their data were analyzed. The baseline characteristics of each group are summarized in Table 1. Participants in the two groups were well-matched in terms of baseline demographics, anthropometric and biochemical characteristics and no significant difference was found between compliant and non-compliant participants. Approximately 62% of the participants were women and the mean age was 32 years.

**Table 1:** Baseline characteristics of the placebo and intervention groups.

Parameter	Normal value	Placebo (n = 27)	Intervention (n = 21)	*p* value
		M ± SD	M ± SD	
Age (years)		31.18 ± 10.06	34.19 ± 11.75	0.345
Gender (n%)				0.202
Male		8 (16.7)	10 (20.8)	
Female		19 (39.6)	11 (22.9)	
Height (m)		1.62 ± 0.08	1.65 ± 0.08	0.158
Weight (kg)		116.38 ± 27.02	120.01 ± 24.45	0.633
Body mass index (kg/m^2)^		43.90 ± 7.32	43.45 ± 6.82	0.829
Excess weight (kg)		50.52 ± 22.71	51.26 ± 20.44	0.907
Muscle mass (kg)		53.30 ± 13.99	59.66 ± 15.37	0.214
Fat mass (kg)		58.68 ± 17.14	56.85 ± 14.76	0.699
Fat percentage(%)		50.27 ± 6.45	47.39 ± 7.73	0.165
Total protein (gm/l)	64–83	70.64 ± 4.68	71.04 ± 4.40	0.764
Albumin (gm/L)	40–150	34.70 ± 3.72	35.04 ± 4.12	0.764
Hemoglobin (gm/dL)		13.07 ± 1.76	12.98 ± 1.84	0.854
Male	13.8–16	14.87 ± 1.78	14.40 ± 1.46	0.544
Female	12.1–16	12.32 ± 1.10	11.69 ± 1.01	0.133
Iron (umol/l)	5.4–28.6	12.75 ± 6.39	13 ± 5.23	0.931
Ferritin (mcg/l)	11–304	29.40 ± 37.19	85 ± 57.93	0.143
Vitamin B12 (pmol/l)	133–675	252.73 ± 108.13	253.73 ± 133.57	0.979
Folate (nmol/l)	4–45	19.67 ± 7.08	18.85 ± 7.62	0.797
Vitamin D (ng/ml)	30–50	17.61 ± 11.41	12.66 ± 7.31	0.196
Calcium (mmol/l)	2.1–2.5	2.23 ± 0.09	2.29 ± 0.11	0.045
Magnesium (mmol/l)	0.72–1.04	0.79 ± 0.07	0.81 ± 0.06	0.482
Zinc (umol/l)	10.1–16.8	13.16 ± 1.83	12.85 ± 1.94	0.651
Copper (umol/l)	11–22	20.34 ± 3.77	19.82 ± 4.52	0.848
Urea (mmol/l)	2.8–8.1	3.36 ± 1.35	3.93 ± 1.01	0.114
Creatinine (umol/l)	53–97	61.33 ± 10.35	64.52 ± 11.47	0.318

SD, standard deviation; n, number; m, meter, independent sample T-test used for comparisons Values in italics mean that comparisons of results are statistically significant.

Table 2 compares the changes in anthropometric and body composition measures between intervention and placebo groups at baseline, 1, 3, and 6 months post LSG. In terms of anthropometric parameters, there was no significant difference in weight, BMI, BMI change, EWL%, TWL%, and absolute weight loss between the two groups. As expected, the weight decreased significantly during the initial 6 months after surgery with EWL% achieved in the intervention group post-LSG being 28.6%, 48.6% and 69.4% at 1, 3 and 6 months, respectively, and for the control group being26.1, 48.4 and 71.4 at 1, 3 and 6 months, respectively. In terms of body compositions, participants in both groups displayed significant improvement in body composition and no statistically significant changes were detected between the two groups in the muscle mass, the percentage loss of muscle mass was noticeably higher in the control group over the 3 timelines. Moreover, fat percentages were less in the intervention group, although the difference wasn't statistically significant except at 1-month post SLG, with 35.2% and 21.8% for placebo and intervention groups, respectively (P < 0.04), although this difference leveled up towards 3 and 6 months follow up. Muscle mass loss accounted for 16.58%, 17.47%, and 20.25% of total weight loss in the intervention group at 1, 3, and 6 months respectively. As anticipated, the majority of weight loss was in the form of body fat % [(89.2%, 82.54%, and 79.27%) in the intervention group at 1, 3 and 6 months, respectively, compared to (80.46%, 76.38%, and 77.58%) in the placebo group at 1, 3 and 6 months respectively]. Although our results show a higher fat % loss in the intervention group, these findings did not reach the statistical significance level.

**Table 2:** Changes in anthropometrics and body composition at 1, 3- and 6-months post-LSG.

	Study Group	Baseline	1 Month	3 Month	6 Month
Weight (kg)	Placebo	116.38 ± 27.02	104.83 ± 25.74***	94.47 ± 24.33***	82.55 ± 23.64***
	Intervention	120.01 ± 24.45	105.20 ± 24***	96.91 ± 20.10***	87.52 ± 18.08***
	*p value*	0.633	0.967	0.715	0.449
Body Mass Index (kg/m^2)^	Placebo	43.90 ± 7.32	39.71 ± 6.98***	35.73 ± 7.03***	31.40 ± 7.42***
	Intervention	43.45 ± 6.82	38.20 ± 5.97***	35.08 ± 5.81***	31.73 ± 5.79***
	*p value*	0.829	0.516	0.735	0.875
BMI change (kg/m^2)^	Placebo		4.73 ± 1.10	8.34 ± 2.12***	12.18 ± 2.67 ***
	Intervention		4.95 ± 1.94	8.37 ± 2.04***	11.91 ± 3.23***
	*p value*		0.665	0.955	0.769
EWL%	Placebo		26.11 ± 7.95	48.37 ± 15.23***	71.40 ± 19.27***
	Intervention		28.60 ± 10.55	48.63 ± 15.08***	69.44 ± 21.99***
	*p value*		0.443	0.955	0.757
TWL%	Placebo		10.78 ± 2.45	19.46 ± 4.60***	28.29 ± 6.04***
	Intervention		11.42 ± 3.67	19.23 ± 3.75***	27.645.97***
	*p value*		0.527	0.854	0.726
Absolute weight loss (kg)	Placebo		12.47 ± 3.44	22.54 ± 6.76***	32.05 ± 8.99***
	Intervention		13.80 ± 5.86	23.11 ± 6.69***	33.72 ± 10.71***
	*p value*		0.395	0.775	0.581
Muscle mass (kg)	Placebo	53.30 ± 13.99	53.04 ± 11.68	49.31 ± 11.03***	48.61 ± 11.15***
	Intervention	59.66 ± 15.37	54.38 ± 15.57*	54.33 ± 11.26**	51.80 ± 10.98***
	*p value*	0.214	0.792	0.137	0.447
Fat mass (kg)	Placebo	58.68 ± 17.14	46.99 ± 14.43***	42.07 ± 15.83***	31.92 ± 14.21***
	Intervention	56.85 ± 14.76	49.11 ± 15.20***	38.28 ± 12.71***	31.70 ± 11.06***
	*p value*	0.699	0.710	0.386	0.962
Fat percentage (%)	Placebo	50.27 ± 6.45	35.27 ± 19.99***	42.18 ± 11.10***	37.41 ± 8***
	Intervention	47.39 ± 7.73	21.84 ± 24.02**	37.59 ± 11.67***	35.71 ± 9.04***
	*p value*	0.168	***0.040***	0.171	0.605
Muscle mass loss (%)	Placebo		17.34 ± 28.86	21.65 ± 16.54*	24.78 ± 16.75
	Intervention		16.58 ± 22.87	17.47 ± 21.36	20.25 ± 6.29 *
	*p value*		0.494	0.459	0.398
Fat mass loss (%)	Placebo		80.46 ± 23.91	76.38 ± 17.86*	77.58 ± 5.72
	Intervention		89.20 ± 36.10	82.54 ± 21.87	79.27 ± 12.19
	*p value*		0.494	0.382	0.739

*SD,* standard deviation; *EWL,* excess weight loss; *TWL*, total weight loss Independent sample *t*-test test used for comparisons. Statistics: *p < 0.05, **p < 0.01, ***p < 0.001, = significantly different compared to baseline value; '*p value*' indicates significant differences between the two intervention groups.

Values in bold italics mean that comparisons of results are statistically significant.

Table 3 demonstrates the changes in biochemical parameters at 1, 3, and 6 months post LSG. The mean level total protein showed a significant increase in the intervention group at 3 months (70.07 ± 3.02 *vs 6*7.26 ± 3.88 gm/L) (*P=* 0.027). Likewise, albumin level was higher in the intervention compared to the placebo group over the whole study period with statistically significantly higher levels at 6 months follow-up [(40.09 ± 2.06 *vs* 37.90 ± 2.84 gm/L) (*P=* 0.036)]. It was also noted that albumin levels were significantly increasing in the intervention group with time-lapse starting the baseline till the 6 months follow up checkpoint (37.07, 38.40, and 40.09 at 1, 3, and 6 months post LSG respectively).

**Table 3:** Changes in proteins and micronutrients at 1, 3- and 6-months post-LSG.

	Study Group	Baseline	1 Month	3 Month	6 Month
Total Protein (gm/l)	Placebo	70.64 ± 4.68	70.25 ± 4.73	67.26 ± 3.88*	69 ± 3.46*
	Intervention	71.04 ± 4.40	68.33 ± 2.34	70.07 ± 3.02	69.76 ± 3.65
	*p value*	0.764	0.280	***0.027***	0.604
Albumin (gm/L)	Placebo	34.70 ± 3.72	35.55 ± 3.57	37.45 ± 4.29*	37.90 ± 2.84
	Intervention	35.04 ± 4.12	37.07 ± 3.54 **	38.40 ± 2.77*	40.09 ± 2.06*
	*p value*	0.764	0.329	0.455	***0.036***
Hemoglobin (gm/dL) - Male	Placebo	14.87 ± 1.78	14.58 ± 1.47	13.50 ± 1.38	13.50 ± 1.38
	Intervention	14.40 ± 1.46	14.70 ± 1.33	14.57 ± 0.94	14.57 ± 0.94
	*p value*	0.544	0.896	0.116	0.317
Hemoglobin (gm/dL) - Female	Placebo	12.32 ± 1.10	12.71 ± 0.62	12.63 ± 1.16	12.63 ± 1.16
	Intervention	11.69 ± 1.01	12.30 ± 1.20	12.23 ± 1.17	12.23 ± 1.17
	*p value*	0.133	0.410	0.434	0.659
Iron (umol/l)	Placebo	12.75 ± 6.39	13.98 ± 4.13	15.13 ± 5.33	14.11 ± 2.81
	Intervention	13 ± 5.23	14.61 ± 7.85	14.84 ± 5.61	17.98 ± 3.97
	*p value*	0.931	0.875	0.892	***0.028***
Vitamin B12 (pmol/L)	Placebo	252.73 ± 108.13	319.61 ± 182.38	263.08 ± 98.70	267.38 ± 84.20
	Intervention	253.73 ± 133.57	338.36 ± 283.35	296.26 ± 242.83	269.81 ± 129.79
	*p value*	0.979	0.847	0.553	0.956
Folate (nmol/l)	Placebo	19.67 ± 7.08	15.80 ± 8.59	12.26 ± 6.27	16.72 ± 8.69
	Intervention	18.85 ± 7.62	19.96 ± 9.10	11.88 ± 3.46	6.66 ± 3.09
	*p value*	0.797	0.424	0.860	***0.027***
Vitamin D (ng/ml)	Placebo	17.61 ± 11.41	15 ± 6.80	21.50 ± 14.33	27.50 ± 13.72 **
	Intervention	12.66 ± 7.31	22 ± 6.57 *	20.26 ± 9.73*	20 ± 12.53
	*p value*	0.196	0.069	0.776	0.219
Calcium (mmol/l)	Placebo	2.23 ± 0.09	2.34 ± 0.08	2.36 ± 0.12	2.34 ± 0.11
	Intervention	2.29 ± 0.11	2.39 ± 0.19	2.36 ± 0.13**	2.41 ± 0.11*
	*p value*	*0.045*	0.533	0.880	0.174
Magnesium (mmol/L)	Placebo	0.79 ± 0.07	0.77 ± 0.06*	0.76 ± 0.05	0.80 ± 0.05
	Intervention	0.81 ± 0.06	0.80 ± 0.07	0.83 ± 0.08	0.80 ± 0.06
	*p value*	0.482	0.391	***0.008***	1.00
Zinc (umol/L)	Placebo	13.16 ± 1.83	12.22 ± 1.76*	12.29 ± 2.70*	11.42 ± 2.10
	Intervention	12.85 ± 1.94	13.12 ± 1.92	13.18 ± 1.79	11.93 ± 3.41
	*p value*	0.651	0.278	0.319	0.728
Copper (umol/l)	Placebo	20.34 ± 3.77	18.70 ± 5.73	18.88 ± 6.92	26.45 ± 11.68
	Intervention	19.82 ± 4.52	19.88 ± 6.41	17.40 ± 2.34	16.44 ± 1.47
	*p value*	0.848	0.774	0.596	0.092
Urea (mmol/l)	Placebo	3.36 ± 1.35	2.68 ± 0.75*	5.73 ± 12.23	3.96 ± 0.65
	Intervention	3.93 ± 1.01	2.92 ± 1.52	3.18 ± 0.79	3.74 ± 1.15
	*p value*	0.114	0.653	0.428	0.547
Creatinine (umol/l)	Placebo	61.33 ± 10.35	57.80 ± 6.62	57.59 ± 9.19	55.75 ± 10.51
	Intervention	64.52 ± 11.47	65.50 ± 9.48	61.40 ± 11.24	63.18 ± 10.81
	*p value*	0.318	0.076	0.266	0.087

*SD,* standard deviation; *EWL*, excess weight loss; *TWL*, total weight loss percentage An independent sample *t*-test was used for comparisons. Statistics: *p < 0.05, **p < 0.01, ***p < 0.001, = significantly different compared to baseline value; '*p value*' indicates significant differences between the two groups.

Values in bold italics mean that comparisons of results are statistically significant.

The intervention group had also seen a significant increase in magnesium levels at 3 months and the iron level at 6 months (*P* = 0.008 *& P=*0.028, respectively). Conversely, the folate level was significantly lower in the intervention group at 6 months compared with the placebo (6.66 ± 3.09 *vs*.16.72 ± 8.69 nmol/l). No other significant differences were detected between the two groups concerning other micronutrients during the entire study period. The renal function remained stable throughout the study period with no significant changes between the two groups.

Dietary intake of the two groups at 3 and 6 months post LSG are shown in Table 4. The proportion of participants consuming a moderate dietary amount of whole meat, eggs, and dairy products as a source of proteins was significantly higher in the intervention group compared to placebo (100% *vs*. 85%, *P=0.05* and 95.2% *vs*. 74.1%, *P=0.051*) at 3 and 6 months respectively. On the other hand, the proportion of participants consuming a low amount of protein-containing food items was significantly higher in placebo compared to the intervention group (14.8% *vs*. 0% and 25.9% *vs*. 4.8%, *P=0.051*) at 3 and 6 months respectively.

**Table 4:** The dietary intake of the two groups at 3 and 6 months post LSG.

		3 Months			6 Months			*p* value		
		Low	Moderate	High	Low	Moderate	High	L vs L	M Vs M	H vs H
Whole meat, eggs &dairy products	Placebo (*n*) %	4 (14.8%)	23 (85.2%)	-	7 (25.9%)	20 (74.1%)	-	0.200	0.200	-
	Intervention (*n*) %	0 (0%)	21 (100%)	-	1 (4.8%)	20 (95.2%)	-	-	-	-
	P	***0.051***	***0.05***	-	***0.051***	***0.051***	-			
Starchy food^a^	Placebo (*n*) %	5 (18.5%)	21 (77.8%)	1 (3.7%)	15 (55.5%)	12 (44.5%)	-	0.825	0.535	-
	Intervention (*n*) %	4 (19%)	17 (81%)	0 (0%)	11 (52.4%)	10 (47.6%)	-	***0.034***	***0.034***	-
	P	0.963	0.788	0.373	0.827	0.827	-			
Fruits & Vegetables	Placebo (*n*) %	2 (7.4%)	24 (88.9%)	1 (3.7%)	7 (25.9%)	17 (63%)	3 (11.1%)	0.419	0.888	0.719
	Intervention (*n*) %	0 (0%)	20 (95.2%)	1 (4.8%)	1 (4.8%)	16 (76.2%)	4 (19%)	-	0.067	***0.035***
	P	0.203	0.430	0.856	***0.051***	0.327	0.440			

*n,* number of participants; Pearson Chi-square test used for comparisons *L vs L,* Low versus Low; *M vs M,* Moderate versus Moderate; *H vs H*, High versus High Values in bold italics mean that comparisons of results are statistically significant.

a Strachey food include pasta, bread, grains and sweet.

We also found that the parentage of participants who consumed a low amount of fruits and vegetables was significantly higher at 6 months in the intervention group *(P=0.051*).

Intake of starchy food was found to be comparable without any significant difference between both groups at three and six months post LSG. Similar results were shown regarding vegetable and fruit intake. Both intervention and placebo groups showed no significant difference and the only statistically significant result was the proportion of patients consuming fruits and vegetables being higher in the placebo group when compared to intervention.

## 5 Discussion

There is a paucity of literature regarding the efficacy of protein supplement post-LSG. This randomized controlled trial demonstrated that protein supplements post-LSG significantly improved mean level of total protein and magnesium at 3 months as well as serum albumin and iron levels at 6 months thereby providing the much-needed nutritional support in the first few months post-LSG. Participants in both groups achieved a significant reduction in weight, BMI, and fat mass while maintaining muscle mass compared with their baseline. While the protein supplement had no statistically significant effect on weight loss or body composition compared to standard dietary plan post LSG, our findings clearly showed trends towards less muscle mass % loss in the intervention group. This finding somewhat represents support for muscle preservation due to protein supplement use. Had we had more participants in the trial, these trends could have possibly reached the statistical significance level. While the number of participants poses such risk, it also highlights the potential of protein supplement with larger sample size.

Our study found no significant differences in mean weight, BMI, BMI change, EWL%, and absolute weight loss between intervention and placebo groups. A similar finding has been reported in a study by Schollenberge and colleagues [30]. However, one study with a longer follow-up duration found that protein supplementation resulted in a significant reduction in BMI in the intervention compared with the non-intervention group (*P* < 0.05) [27]. As expected, participants in both groups lost significant weight at 1,3 and 6 months post LSG which was accompanied by significant modification of body composition. Other studies supported our findings. Maïmoun et al. [5] found that as early as one-month post-surgery, the acute weight loss in LSG resulted in significant muscle mass loss (*P <0.001).*

There was an increase in the muscle mass in the intervention compared to the placebo group throughout the study period. However, these changes were not statistically significant (P = 0.792, P = 0.137, P = 0.447 respectively). Likewise, the total body weight loss attributed to the muscle mass loss was slightly lower in the intervention group throughout the study period, although it didn't reach statistical significance. lack of statistical significance in muscle mass and percentage of muscle mass loss between the two groups may be related to the small sample size. Our results are in accordance with Moize et al. [29] who showed that daily protein supplement was associated with better retention of months muscle mass at 4 and 12 months post-bariatric surgery (*P* < 0.001 & *P< 0.031* respectively) emphasizing on the importance of protein supplements during fast weight loss [29]. Maintaining adequate muscle mass is important because muscles play a central role in body protein metabolism, resting metabolic rate (RMR), and weight loss [[7], [8], [9]]. Most of the weight loss in our intervention and placebo groups was attributed to a fat loss where the maximum fat loss achieved as early as the first month in the placebo group (89.2%) and at 3 months in the intervention group (82.54%).

One important finding of our study is that the total protein level at 3 months was significantly higher in the intervention group compared with placebo thereby warranting that greater protein intake would counteract the catabolic effect of bariatric surgery. This finding is in contrast with previous studies where there was no significant difference between participants who received protein supplements and placebo groups [30]. Similarly, Albumin level at 6 months was significantly higher in the intervention than the placebo group (40.09 ± 2.06 *vs.* 37.90 ± 2.84 gm/L, *P=0.036*). Overall, these results reinforce the importance of protein supplements in preventing essential protein deficiency. An observational study among LSG and RYGB found that in subjects achieving higher protein intake using protein supplements, the plasma albumin did not change significantly at 4, 8, and 12 months compared with their baseline [13].

Our results also showed significantly higher levels of serum iron at 6 months in the intervention group compared with the placebo (*P* = *0.028*). This finding is in contrast with other reports where the iron level remained relatively stable and did not differ significantly with a protein supplement [28,30]. Our data support that protein supplement not only provide nutritional support but also may help to maintain iron reserve post-LSG. Iron plays an important role in erythropoiesis and the depletion of body iron stores may result in anemia. The level is negatively affected post LSG due to intolerant of red meats which is a vital source of dietary iron and also because of decreased absorption associated with reduced gastric acid necessary for absorption [30]. Likewise, our study found a magnesium level to be significantly higher in the intervention than that of the placebo at 3 months. In contrast, previous research found that magnesium level did change significantly at 6 months in patients taking micronutrient supplements compared with patients who are not compliant with supplements [34]. An interesting finding in our study was that folate was significantly lower in the intervention group at 6 months (*P=0.027)*. Other author*s* found that folate deficiency post-LSG was decreased from 18.2% to 8.8% with supplementation [28]. Decreased levels of folate may result from poor dietary intake of folate-rich food or nonadherence to multi-vitamins supplement. Deficiency of folate can cause megaloblastic anemia, cognitive impairment, and risk for congenital neural tube defects [10,18,35].

Several studies reported significant changes in dietary intake especially in the first 6 months post-LSG [13,28]. Our assessment of macronutrient intake using a food frequency questionnaire confirmed such findings. We observed that the percentage of participants consuming moderate dietary protein at 6 months was higher in the intervention group compared with placebo (95.2% *vs*. 74%, *P=0.051).* Ensuring adequate protein intake has been shown to improve the efficiency of bariatric surgery in terms of weight loss and improvement in body composition [19,26]. One study demonstrated that greater protein intake after RYGB was associated with lower consumption of carbohydrate which is likely due to the increased satiety observed with protein intake [26]. Conversely, we found that the percentage of participants consuming a low amount of protein was significantly higher in placebo compared with the intervention group (25.9% *vs*. 4.7%). This is consistent with other research where 58.3% of patients (with no protein supplement) had low protein intake at 4 months post-bariatric surgery [13]. Moreover, studies also found insufficient protein intake was more pronounced in patients with protein intolerance post-surgery [13,16]. Overall, these results reinforce the importance of the addition of protein supplements to the post-bariatric diet is to provide bariatric patients with the necessary protein to alleviate postsurgical protein loss. We also found that the parentage of participants who consumed a low amount of vegetables and fruits was significantly higher at 6 months placebo than the intervention group in *(P=0.051).* This is consistent with other authors who observed a decrease in vegetables and fruits shorty after surgery (1 month) with a gradual increase thereafter. Low intake of vegetables and fruits may affect the adequacy of fiber and soluble vitamins. Therefore, a dietitian should encourage bariatric patients to increase their consumption of vegetables and fruits to avoid vitamin deficiency.

### 5.1 Study limitations

We acknowledge that our study has some important limitations. Firstly, the placebo group was given a solution that contained 100 calories compared to 250 calories in the intervention group. Such a difference might represent a disadvantage to the intervention group consuming a higher calorie solution. Secondly, we didn't report daily energy intake and we used food frequency to assess macronutrient intake instead of more precise methods such as 3-day food records and 24-h dietary recall. We monitored the adherence of the participants to the treatment over the phone, which arguably makes the compliance of the participants questionable, however, participants were encouraged to use diaries to register their daily supplement use. Thirdly, the use of bioelectrical impedance for assessment of body composition in individuals with obesity, although is practical in clinical setting and has comparable results to dual-energy x-ray [36], it may have overestimated muscle mass [37]. Fourthly, intention to treat analysis was not performed following the more than expected loss to follow-up, primarily due to our strict compliance policy that saw a number of participants excluded from the study. The low number of the endpoint of trial may also be seen as a strength as some of our findings that showed trends or failed to reach statistical significance could have been achieved the statistical significance in relation to some of the fundamental findings such as muscle mass % etc.

Although we had some statistically significant findings in this trial, these preliminary results need to be confirmed by larger RCT. A larger study may further confirm some of the results that did not reach statistical significance in our study.

The strength of our study is that this is the first RCT to examine the effect of protein supplements among LSG patients. We assessed a range of parameters including anthropometrics, body composition, and a range of micronutrients. Our findings add a body of evidence to the existing literature that advocates the use of protein post-bariatric surgery and may help to establish guidelines regarding role protein supplement in preventing protein deficiencies post LSG.

## 6 Conclusion

Our study shows that protein supplement post-LSG significantly improved total protein, albumin, iron, and magnesium, although it has no significant impact on weight, muscle mass, or fat mass loss. However, the protein supplement helped in maintaining muscle mass and preventing muscle mass loss. Further studies with larger sample size are warranted to evaluate the effectiveness of protein supplement in promoting weight loss, preserving muscle mass, and reducing the risk of developing protein malnutrition post-bariatric surgery.

## Ethical approval

All procedures performed in this study were in accordance with the ethical standards of the institutional and/or national research committee and with the 1964 Helsinki Declaration and its later amendment or comparable ethical standards.

## Funding

There was no funding for this research.

## Author contribution

SS, MAS and FH developed the study concept, study design, data analysis and interpretation as well as writing and final revision of the paper.

SS contributed to data collection and supervision of research nurses.

LA, HA, WH contributed to study concept, data interpretation and revision of the manuscript.

## Informed consent

Informed consent was waived (IRB approved, HIPAA compliant retrospective study).

## Registration of research studies

1. Name of the registry: http://www.researchregistry.com
2. Unique Identifying number or registration ID: (researchregistry7348).
3. Hyperlink to your specific registration (must be publicly accessible and will be checked): https://www.researchregistry.com/browse-the-registry#home/?view_2_search=researchregistry7348&view_2_page=1

## Guarantor

SS, MAS and FH.

## Provenance and peer review

Not commissioned, externally peer-reviewed.

## Declaration of competing interest

All authors declare no Conflict of interest.
